# Prolonged COVID 19 Outbreak and Psychological Response of Nurses in Italian Healthcare System: Cross-Sectional Study

**DOI:** 10.3389/fpsyg.2021.608413

**Published:** 2021-04-06

**Authors:** Jessica Ranieri, Federica Guerra, E. Perilli, Domenico Passafiume, D. Maccarone, C. Ferri, Dina Di Giacomo

**Affiliations:** ^1^Life, Health and Environmental Sciences Department, University of L'Aquila, L'Aquila, Italy; ^2^S. Salvatore Hospital, L'Aquila, Italy

**Keywords:** clinical psychology, nurse, COVID-19, psychological traits, personality traits, healthcare worker

## Abstract

Aim of the study was to analyze the posttraumatic stress disorder risk nurses, detecting the relationship between distress experience and personality dimensions in Italian COVID-19 outbreak. A cross-sectional study was conducted based on 2 data detection (March 2020 and September 2020). Mental evaluation was carried out in Laboratory of Clinical Psychology on n.69 nurses in range age 22–64 years old (mean age 37.3; sd ± 10.3; 55% working in nursing care with confirmed COVID-19 patients (named frontline; secondline nurses have been identified by nursing care working with infectious patients but no confirmed COVID-19). Measurement was focused on symptoms anxiety, personality traits, peritraumatic dissociation and post-traumatic stress for all participants. No online screening was applied. Comparisons (ANOVA test) within the various demographic characteristics demonstrated few significant differences between groups on DASS-21, PDEQ, and ISE-R scores. Correlation analysis (Spearman test) was performed among PDEQ, DASS-21, BFI-10 and IES-R and confirmed between anxiety (DASS-21) and peritraumatic dissociation and post-traumatic stress; then anxiety is positively correlated to agreeableness variable of BFI-10 test. The emotional distress was protracted overtime (after 6 months) but in long-term personality traits resulted mediator facing subjective stress. Our finding drew details for protective and predictive risk factors as well as mental health issues of nurses dealing with pandemic: healthcare workers faced the protracted challenge caring COVID-19 patients over and over again: in short time the impact was relevant, and the prolonged exposition to the stressor was tackled by personal resources such as personality traits.

## Introduction

Actual COVID-19 pandemic is affecting mental health of population. Several mental health screening were conducted for general population (Fan et al., 2021; Simşir et al., 2021), undergraduate, children and adolescent as well-health care workers (Lai et al., 2020; Ranieri et al., 2020; Rossi et al., 2020; Seçer et al., 2020; Wang et al., 2020). Depression, anxiety, peritraumatic dissociative experience and mental disease were detected as response in acute COVID-19 outbreak; healthcare workers, in particular young women and frontline, resulted intensively affected (Zhang et al., 2020).

Most researches conducted in coronavirus pandemic have been conducted applying survey method trying to involve the higher number of health care workers, as well general population in order to have the preliminary data about impact of pandemic on mental health; they screened mental health population detecting a general mental suffering. The limitation of those studies is into the application of survey methodology: it is evident the lacking for gold standard for psychological evaluation setting, so the results could be exposed to the lacking of objectivity; more, applying online survey potential bias effect could be the timing of completion online form being a web link available to everyone and exposed to potential risk for reliability data; last but not least, the lacking of demographic data related to the mental health preview pandemic (personal history of individuals) detectable by anamnesis and clinical interview as well the use of psychotropic drugs.

Moreover, several studies were based on emotional and well-being self-perception in short-time screening session. Prolonged pandemic exposition and the spread out of mental health needs are demanding to draw the effective psychopathological impact on mental health in health workers applying gold standard for mental health measurement and longitudinal as well as cross-sectional study designs.

February 2020 period started the Italian acute COVID-19 outbreak and stressing the healthcare system in terms of management of hospitalization procedure and management of emotional impact on healthcare workers in hospital emergency worried about the risk for own health (Protezione Civile, 2020). According Lai's et al. finding (2020), we wanted to evaluate the mental health of healthcare workers in short- and long-term Italian COVID-19 outbreak applying psychological measurements for posttraumatic disorder risk in traditional psychological setting applying a cross-sectional study design based on early (short-) and prolonged (long-) time of outbreak. Aim of the study was to investigate the posttraumatic stress disorder risk in healthcare workers facing protracted challenge of coronavirus phenomena, detecting the relationship between distress experience and personality dimensions.

## Methods

### Ethical Approval

This study was approved by the Internal Review Board (IRB) of the University of L'Aquila, Italy (Prot. No. 107751/2020). Written informed consent was obtained following the Helsinki Declaration (WMA).

### Study Design

Participants have been enrolled in Clinical Psychology Laboratory of University of L'Aquila. Informed consent was obtained from each participant at the time of enrolment and the study adhered to the Declaration of Helsinki. The study is cross-sectional study based on mental screening conducted in March 2020 and in September 2020. During March period, the total confirmed cases of COVID-19 exceeded 101.739 in Italy, whereas in September confirmed cases were 314.861 (Open Data). Trained clinical psychologists, blind to the objectives of the study, conducted the psychological screening in a quiet, dedicated room. The duration of the evaluations was 45 min. Data were collected anonymously.

### Participants

Eligible participants were nurses aged 22–64 years old (mean age 37.3; sd ± 10.3). Demographic characteristics of the n.69 participants are: 49.2% (n.34) of them were married, 62.3% having no children; 55% working in nursing care with confirmed COVID-19 patients (named frontline; secondline nurses have been identified by nursing care working with infectious patients but no confirmed COVID-19). Inclusion criteria were: (a) aged 22–65; (b) being female; (c) being nurses in National Healthcare system; (d) no signs for previous psychological disorders and/or chronic disease.

### Outcomes

Demographic data were self-reported by participants. Measurement was focused on symptoms anxiety, personality traits, peritraumatic dissociation and posttraumatic stress for all participants. Psychological battery has been composed of n.4 self-reports evaluating the anxiety (DASS-21), personality traits (BFI-10) and distress (EIS-R and PDEQ) to measure the presence/absence of psychological symptoms and related severity.

#### Big Five Inventory-10

The BFI-10 (Guido et al., 2015) evaluates the five personality dimensions on a 5-point scale ranging from 1 (strongly disagree) to 5 (strongly agree), each with two items: openness (OP), conscientiousness (CO), emotional stability (ES), extraversion (EX), and agreeableness (AG).

#### Depression, Anxiety, and Stress Scale (DASS-21)

The DASS (Beaufort et al., 2007) is a clinical assessment that measures the three related states of depression, anxiety and stress. It has 21 questions and takes about 3 min to complete. Each subscale measuring the emotional traits is composed of 7 items. We applied only the anxiety subscale.

#### Impact of Event Scale-Revised

It is a 22-item self-report questionnaire (Marmar et al., 1997) to measure the subjective response to a specific traumatic event, especially in the response sets of intrusion (intrusive thoughts, nightmares, intrusive feelings and imagery, dissociative-like re-experiencing), avoidance (numbing of responsiveness, avoidance of feelings, situations, and ideas), and hyperarousal (anger, irritability, hypervigilance, difficulty concentrating, heightened startle), as well as a total subjective stress IES-R score. Scores higher than 33 are of concern; the higher the score the greater the concern for post-traumatic stress and associated health and well-being consequences.

#### Peritraumatic Dissociative Experiences Questionnaire

It is a 10-items self-report questionnaire (Weiss, 2007) measuring peritraumatic dissociation. The PDEQ has well-established psychometric properties, with higher total scores indicating increased peritraumatic dissociation. A score above 15 is indicative of significant dissociation.

### Statistical Analyses

The data analysis was performed using SPSS statistical software, with a fixed α ≤ 0.05. All demographic data were analyzed and presented as number (N) and percentage (%). Using MANOVA test as appropriate, we compared emotional severity by demographic variables. Spearman rank order correlation was used to examine correlations among anxiety, peritraumatic, post-traumatic stress and psychological traits.

## Results

### Analysis of Emotional Dimension in Early Italian Outbreak (March 2020)

First, we analyses the prevalence of emotional symptoms among nurses in early time of outbreak (March 2020) (Table 1).

**Table 1:** Raw score of anxiety, distress, and PT stress symptoms in the sample in March 2020 detection data.

	**Age group**		**Marital status**		**Level Caring**	
	**Younger (X, sd)**	**Old (X, sd)**	**Married (X, sd)**	**Single (X, sd)**	**Frontline (X, sd)**	**Secondline (X, sd)**
DASS-21 anxiety	6.6 ± 3.7	4.4 ± 1.8	4 ± 2.3	6.5 ± 3.1	5.4 ± 3.4	5.8 ± 1.6
PDEQ	19.9 ± 8.3	15.8 ± 3.8	15.5 ± 3.6	19.4 ± 7.8	18.4 ± 7.4	16.2 ± 3.6
BFI-10 AG	5.4 ± 1.9	5.5 ± 1.5	5.2 ± 1.1	5.6 ± 2	5.3 ± 1.8	5.8 ± 1.5
BFI-10 CO	7.7 ± 1.6	8.4 ± 1.6	8.4 ± 1.3	7.8 ± 1.8	8 ± 1.6	8.2 ± 1.9
BFI-10 ES	6.1 ± 1.1	6.1 ± 1.8	6.2 ± 1.7	6 ± 1.3	5.9 ± 1.1	6.7 ± 2.2
BFI-10 EX	6.4 ± 2.5	6 ± 1.5	6.1 ± 1.9	6.3 ± 2.2	6.3 ± 2.2	5.7 ± 1.6
BFI-10 OP	6.5 ± 1.9	6.8 ± 1.8	6.5 ± 1.7	6.7 ± 2	6.7 ± 1.9	6.6 ± 1.8
IES-R avoidance	13.7 ± 7.1	10.6 ± 5	9.3 ± 5.2	14 ± 6.3	12.5 ± 6.9	11.1 ± 4
IES-R intrusivity	14.5 ± 7.3	12.6 ± 6.8	10.4 ± 6.1	15.6 ± 7	13.4 ± 7.2	14.1 ± 6.9
IES-R iperarousal	10.5 ± 6.1	8.3 ± 4.9	6.6 ± 4.7	11.2 ± 5.5	9.3 ± 5.7	9.7 ± 5.7
IES-R TOT	38.8 ± 19.4	31.4 ± 15.5	26.3 ± 14.6	40.9 ± 17.5	35.2 ± 18.6	34.9 ± 15.6

*X, mean value*.

A considerable part of sample (77.3%) showed anxiety: 10.5% extremely severe, 13.1% severe, 28.9% moderate, and 23.7% mild level [DASS-21 subscale Anxiety >8 (score range 0–21)]. Fifty five percent of the sample evidenced significant peritraumatic dissociative experience [PDEQ score >15 (score range 1–50)] as well as 52.6% of nurses showed a probable presence of post-traumatic stress [IES-R score >33 (score range 0–88)] and 47.3% resulted in no stressed emotional condition.

For dimensions of personality status, the prevalence for each categories were: 79% high level of conscientiousness (21% moderate, no low level); 57, 9% moderate level of emotional stability (28.9% high and 13.1% low level); 50% moderate level of openness (39.4% high and 10.5% low level); 44.7% moderate extroversion level (36.8% high and 18.4% low level); then, 42.1% moderate level of agreeableness (34.2% low and 23.6% high level).

Comparisons (ANOVA test) within the various demographic characteristics demonstrated few significant differences between groups on DASS-21, PDEQ, and ISE-R scores. By age groups (median value = 35 years old) younger nurses showed higher anxiety (DASS-21) then old group (η = 0.6; *p* = 0.02). Marital status resulted significant: single nurses evidenced higher anxiety than married (η = 0.7; *p* = 0.01), as well as single reported higher level of post-traumatic stress than married ((η = 0.7; *p* = 0.01). Nursing care wasn't significant.

Correlation analysis (Spearman test) was performed among BFI-10, PDEQ, DASS-21, and IES-R. The results summarized in Table 2 confirm between anxiety (DASS-21) and peritraumatic dissociation and post-traumatic stress; then anxiety is positively correlated to agreeableness variable of BFI-10 test. Last, subjective stress for events (IES-R) was related to the anxiety (*p* = 0.001) and peritraumatic dissociation dimension (*p* = 0.001) for all indexes (avoidance, intrusivity, and hyperasoul). No correlation was significant by personality traits and emotional dimensions.

**Table 2:** Spearman correlations among emotional and psychological measurements in March 2020 evaluations correlation matrix.

		**PDEQ**	**DASS-21 anxiety**	**BFI-10 AG**	**BFI-10 CO**	**BFI-10 OP**	**BFI-10 EX**	**IES-R avoidance**	**IES-R intrusivity**	**IES-R iperarousal**	**IES-R TOT**
PDEQ	Spearman's rho	–									
	*p*-value	–									
DASS-21 anxiety	Spearman's rho	0.521^***^	–								
	*p*-value	<0.001	–								
BFI-10 AG	Spearman's rho	0.341^*^	0.249	–							
	*p*-value	0.036	0.131	–							
BFI-10 CO	Spearman's rho	−0.229	−0.132	0.102	–						
	*p*-value	0.167	0.431	0.543	–						
BFI-10 OP	Spearman's rho	−0.019	−0.042	0.086	0.402^*^	–					
	*p*-value	0.912	0.804	0.608	0.012	–					
BFI-10 EX	Spearman's rho	0.048	−0.159	0.019	0.281	−0.003	–				
	*p*-value	0.776	0.340	0.911	0.087	0.985	–				
IES-R avoidance	Spearman's rho	0.637^***^	0.374^*^	0.081	−0.084	0.280	−0.019	–			
	*p*-value	<0.001	0.021	0.630	0.614	0.088	0.908	–			
IES-R intrusivity	Spearman's rho	0.578^***^	0.484^**^	0.169	−0.110	0.274	0.017	0.783^***^	–		
	*p*-value	<0.001	0.002	0.310	0.511	0.096	0.918	<0.001	–		
IES-R iperarousal	Spearman's rho	0.663^***^	0.496^**^	0.015	−0.106	0.272	−0.062	0.776^***^	0.777^***^	–	
	*p*-value	<0.001	0.002	0.929	0.527	0.099	0.712	<0.001	<0.001	–	
IES-R TOT	Spearman's rho	0.692^***^	0.504^**^	0.140	−0.105	0.310	−0.015	0.917^***^	0.926^***^	0.904^***^	–
	*p*-value	<0.001	0.001	0.401	0.530	0.058	0.929	<0.001	<0.001	<0.001	–

* *p < 0.05*,

** *p < 0.01*,

*** *p < 0.001*.

### Analysis of Emotional Dimension in Prolonged Italian Outbreak (September 2020)

In Table 3 were reported the raw score of psychological performance of nurses in prolonged outbreak (September 2020).

**Table 3:** Raw score of anxiety, distress, and PT stress symptoms in the sample in September 2020 detection data.

	**Age group**		**Marital status**		**Level caring**	
	**Younger (X, sd)**	**Old (X, sd)**	**Married (X, sd)**	**Single (X, sd)**	**Frontline (X, sd)**	**Secondline (X, sd)**
DASS-21 anxiety	29.6 ± 6.3	23 ± 4.8	26.8 ± 6.2	25.1 ± 6.9	27.4 ± 6.4	24.7 ± 6.4
PDEQ	21.7 ± 8.6	18.4 ± 3.8	21.2 ± 8.0	18.1 ± 7.4	20.0 ± 7.6	20 ± 8.3
BFI-10 AG	6.1 ± 0.7	6.2 ± 1.4	6.3 ± 1.1	5.9 ± 1.2	5.8 ± 1.1	6.6 ± 1.0
BFI-10 CO	8.0 ± 1.9	8.8 ± 1.1	8.0 ± 1.7	9.0 ± 1.1	8.4 ± 1.5	8.4 ± 1.7
BFI-10 ES	5.5 ± 1.0	5.2 ± 1.8	5.4 ± 1.5	5.2 ± 1.4	4.7 ± 1.2	6.1 ± 1.5
BFI-10 EX	6.6 ± 2.5	6.0 ± 2.5	5.8 ± 2.8	7 ± 1.8	6.6 ± 2.4	5.9 ± 2.6
BFI-10 OP	6.1 ± 2.3	7.0 ± 1.4	6.5 ± 2.2	6.5 ± 1.3	7.2 ± 1.9	5.7 ± 1.6
IES-R avoidance	13.6 ± 6.9	11.6 ± 6.6	12.5 ± 7.7	12.8 ± 5.1	12.2 ± 7.0	13.0 ± 6.6
IES-R intrusivity	14.6 ± 6.2	12.3 ± 6.9	12.7 ± 7.1	14.5 ± 5.9	12.9 ± 6.3	14.0 ± 7.1
IES-R iperarousal	12.7 ± 6.3	7.4 ± 4.1	9.8 ± 6.8	10.2 ± 4.4	9.5 ± 4.8	10.4 ± 9.5
IES-R TOT	40.9 ± 18.9	31.4 ± 16.5	35.1 ± 20.4	37.4 ± 14.4	34.8 ± 16.3	37.4 ± 20.5

All of the sample showed anxiety: 87% extremely severe, and 12.9% severe level [DASS-21 subscale Anxiety >8 (score range 0–21)]. Sixty one percent of the sample evidenced significant peritraumatic dissociative experience [PDEQ score >15 (score range 1–50)] as well as 61.2% of nurses showed a probable presence of post-traumatic stress [IES-R score >33 (score range 0–88)] and 38.7% resulted in no stressed emotional condition.

For dimensions of personality, the prevalence for each categories were: 74.1% high level of conscientiousness (25.8% moderate and no low level); 67.7% moderate level of emotional stability (29% low and 3.2% high level); 51.6% moderate level of openness (35.4% high and 12.9% low level); 38.7% moderate extroversion level (35.4% high and 25.8% low level); then, 87% moderate level of agreeableness (9.6% low and 3.2% high level).

Comparisons (ANOVA test,) within the various demographic characteristics demonstrated few significant differences between groups on DASS-21, PDEQ, and ISE-R scores. By age groups (median value = 37 years old) younger nurses showed higher anxiety (DASS-21) then old group (η^2^ = 0.22; *p* = 0.01]. Geographical area of work was significant for anxiety [*F*_(2,1)_ = 5.12; η^2^ = 0.26; *p* = 0.01), peritraumatic dissociative experience [*F*_(2,1)_ = 5.42; η^2^ = 0.27; *p* = 0.01] and probable presence of post-traumatic stress [*F*_(2,1)_ = 3.48; η^2^ = 0.19; *p* = 0.04]; in *post-hoc* analysis (Tukey test), per each measurement was significant the comparison between North and South Italian area evidencing the higher negative emotional outcome in nurses working in North Italy (anxiety *P*_*tukey*_ = 0.009 Cohen's *d* = 1.38; peritraumatic experience *P*_*tukey*_ = 0.007 Cohen's *d* = 1.42; probable presence of post-traumatic stress symptoms *P*_*tukey*_ = 0.03 Cohen's *d* = 1.13). Marital status, nursing care (frontline/secondline) resulted no significant.

Correlation analysis (Spearman test) was performed among BFI-10, PDEQ, DASS-21, and IES-R. The results summarized in Table 4: the correlation between anxiety (DASS-21) and peritraumatic dissociation and post-traumatic stress have been confirmed as well in March detection data; then even the correlation between peritraumatic stress, anxiety, and subjective stress for event have been detected; furthermore, personality traits were correlated negatively with subjective stress, in particular optimism with intrusivity index (*p* = 0.004), extroversion with avoidance (*p* = 0.001) and hyperarousal (*p* = 0.001) indexes.

**Table 4:** Spearman correlations among emotional and psychological measurement in September 2020 evaluations.

		**PDEQ**	**DASS-21 anxiety**	**BIG-10 AG**	**BIG-10 CO**	**BIG-10 ES**	**BIG-10 OP**	**BIG-10 EX**	**IES-R avoidance**	**IES-R intrusivity**	**IES-R Iperarousal**	**IES-R TOT**
PDEQ	Spearman's rho	–										
	*p*-value	–										
DASS-21 anxiety	Spearman's rho	0.614^***^	–									
	*p*-value	<0.001	–									
BIG-10 AG	Spearman's rho	0.162	−0.027	–								
	*p*-value	0.384	0.885	–								
BIG-10 CO	Spearman's rho	−0.037	−0.033	0.162	–							
	*p*-value	0.845	0.859	0.382	–							
BIG-10 ES	Spearman's rho	0.275	0.034	0.084	−0.231	–						
	*p*-value	0.134	0.854	0.654	0.212	–						
BIG-10 OP	Spearman's rho	−0.333	−0.286	0.063	−0.220	−0.369^*^	–					
	*p*-value	0.067	0.119	0.736	0.233	0.041	–					
BIG-10 EX	Spearman's rho	−0.278	−0.189	−0.190	0.453^*^	−0.090	−0.019	–				
	*p*-value	0.130	0.309	0.306	0.010	0.629	0.921	–				
IES-R avoidance	Spearman's rho	0.733^***^	0.615^***^	0.168	−0.091	0.043	−0.312	−0.494^**^	–			
	*p*-value	<0.001	<0.001	0.366	0.627	0.818	0.087	0.005	–			
IES-R intrusivity	Spearman's rho	0.731^***^	0.598^***^	−0.229	0.086	0.128	−0.502^**^	−0.246	0.740^***^	–		
	*p*-value	<0.001	<0.001	0.215	0.646	0.494	0.004	0.182	<0.001	–		
IES-R iperarousal	Spearman's rho	0.784^***^	0.753^***^	−0.025	−0.148	0.169	−0.352	−0.393^*^	0.842^***^	0.858^***^	–	
	*p*-value	<0.001	<0.001	0.894	0.427	0.365	0.052	0.029	<0.001	<0.001	–	
IES-R TOT	Spearman's rho	0.820^***^	0.701^***^	−0.056	−0.050	0.141	−0.477^**^	−0.360^*^	0.883^***^	0.945^***^	0.953^***^	–
	*p*-value	<0.001	<0.001	0.764	0.788	0.451	0.007	0.047	<0.001	<0.001	<0.001	–

*Correlation matrix*.

* *p < 0.05*,

** *p < 0.01*,

*** *p < 0.001*.

## Discussion and Conclusions

This cross-sectional study was focused on the screening of mental health in nurses in 2 differential timing of Italian COVID-19 outbreak; we wanted to investigate the mechanisms of mental adaption to environmental stressor in long-term challenge pandemic. Younger nurses were affected and, other demographic variables were incising in first time marital status was influencing the emotional condition of healthcare worker; afterward, geographical area of work resulted preeminent. Frontline/secondline nursing care was no decisive for emotional impact. Facing COVID-19 patients, healthcare workers developed in early time anxiety symptoms (from extremely severe to mild level) related to peritraumatic dissociative experience and probable sign for posttraumatic stress symptoms related even to subjective stress though intrusive thoughts, avoidance of feelings, situations, and ideas, anger, irritability, hypervigilance, difficulty concentrating. Personality dimension related was the agreeableness based on positive feeling, sincere and trusting.

After 6 months, anxiety for peritraumatic dissociative experiences resulted still effective as well subjective stress; an interesting point was no direct correlation with personality traits.

**Figure 1:**
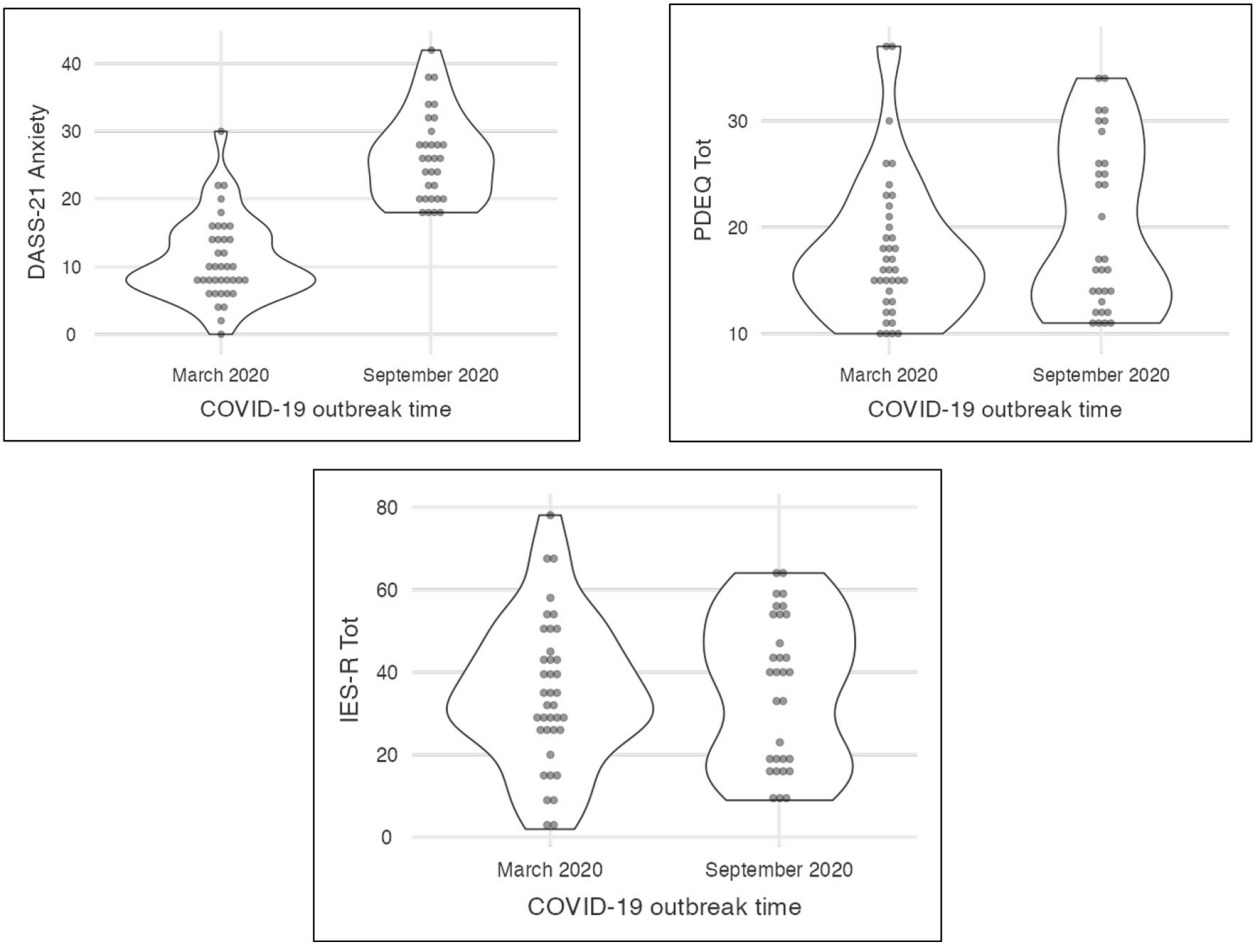
Representation of emotional dimensions in short- and long-term COVID 19 outbreak.

Our finding was obtained in short- and long-term Italian COVID-19 outbreak; we measured its short-time mental health impact for healthcare workers highlighting the anxiety as early reaction for emotional distress and high risk for posttraumatic stress disorders; the personality dimensions haven't mediated the emotional distress as well as probable risk for posttraumatic stress symptoms: the impact of pandemic event on mental health of nurses was strong and unmanageable by themselves; individual resources did not help professionals to overcome the distress. Nurses appeared exposed to mental distress. The emotional distress was protracted overtime (after 6 months) but in long-term personality traits resulted mediator facing subjective stress (see Figure 1).

Our finding drew details for protective and predictive risk factors as well as mental health issues of nurses dealing with pandemic. Several study (Lai et al., 2020; Rossi et al., 2020; Wang et al., 2020) conducted a mental screening in public health emergency and outlined risk trend for health workers; our study has confirmed and implemented findings. Healthcare workers faced the protracted challenge caring COVID-19 patients over and over again: in short time the impact was relevant, and the prolonged exposition to the stressor was tackled by personal resources such as personality traits. Our findings highlighted in short time the intensive impact of COVID 19 outbreak in healthcare workers, as well as reported in recent literature (Lai et al., 2020; Ranieri et al., 2020; Wang et al., 2020), and in long time they have resorted to their own psychological resource (personality traits) providing the personal adaptation to the environmental prolonged outbreak stressor, toward to mitigate the negative effect of actual pandemic on mental health. Results evidenced the need to carry on mental health program for health workers (especially nursing professionals) in order to prevent burn out or post-traumatic stress symptoms in who took care of patients in acute COVID-19 outbreak. Moreover, tailored individual programs for empowerment and health promotion strategies might be priority for policy in National Healthcare System toward improvement of Health policy and service (Di Giacomo, 2020). Challenge is going to overcome and steer their mental health risk turning it as protecting process in public health emergency.

The study presents some limitations: the sample size and psychological measurements. Simple size is limited to consent generalization data but could be representative of extensive researches will be realized progressively; then, the psychological measurements are composed of short and fast battery but it was applied in traditional setting reflecting golden standard of psychological testing.

## Data Availability Statement

The original contributions presented in the study are included in the article/supplementary material, further inquiries can be directed to the corresponding author/s.

## Ethics Statement

The studies involving human participants were reviewed and approved by University of L'Aquila IRB (Prof. 37589/2021). The patients/participants provided their written informed consent to participate in this study.

## Author Contributions

DD had full access to all of the data in the study and take responsibility for the integrity of the data and the accuracy of the data analysis. FG, JR, DP, EP, and DM: acquisition, analysis, or interpretation of data. DD: drafting of the manuscript. CF: supervision. All authors: concept and design.

## Conflict of Interest

The authors declare that the research was conducted in the absence of any commercial or financial relationships that could be construed as a potential conflict of interest.
